# Artificial Intelligence in NAFLD: Will Liver Biopsy Still Be Necessary in the Future?

**DOI:** 10.3390/healthcare11010117

**Published:** 2022-12-30

**Authors:** Lei Zhang, Yilei Mao

**Affiliations:** Department of Liver Surgery, Peking Union Medical College Hospital, Chinese Academy of Medical Sciences and Peking Union Medical College, Beijing 100730, China

**Keywords:** artificial intelligence, nonalcoholic fatty liver disease, nonalcoholic steatohepatitis, diagnosis

## Abstract

As the advanced form of nonalcoholic fatty liver disease (NAFLD), nonalcoholic steatohepatitis (NASH) will significantly increase the risks of liver fibrosis, cirrhosis, and HCC. However, there is no non-invasive method to distinguish NASH from NAFLD so far. Additionally, liver biopsy remains the gold standard to diagnose NASH, which is not appropriate for routine screening. Recently, artificial intelligence (AI) is under rapid development in many aspects of medicine. Additionally, the application of AI in clinical information may have the potential to diagnose NASH non-invasively. This review summarizes the latest research using AI, specifically machine learning, to facilitate the diagnosis, prognosis, and monitoring of NAFLD. Additionally, according to our prior results, this work proposes future development in this area.

## 1. Introduction

About 25% of individuals with low alcohol intake were affected by liver disease in a worldwide sample [1]. It has been pointed out that 33.9% of the Asian population had nonalcoholic fatty liver disease (NAFLD) during 2012–2017 [2]. Non-alcoholic steatohepatitis (NASH) is a progressive state of non-alcoholic fatty liver disease. It significantly increases the risks of hepatocellular carcinoma, cirrhosis, and liver fibrosis [3]. To our knowledge, as the most reliable way to diagnose NASH and evaluate hepatic fibrosis stage, liver biopsy is not appropriate for routine screening. The liver biopsy also has certain limitations, including patient acceptability, puncture risk, economic cost, diagnostic heterogeneity, etc. Therefore, non-invasive diagnostic methods to distinguish NASH from NAFLD have important application prospects. 

Artificial intelligence (AI) has been developing rapidly in many aspects of medicine in recent years, including Image recognition, non-invasive diagnosis, and treatment decision making [4,5]. Additionally, the use of AI in clinical information may have the potential to diagnose NASH non-invasively. In this review, we have summarized the latest research using AI to facilitate the diagnosis, prognosis, and monitoring of NAFLD/NASH. We searched for reported studies of human experiments in English till Jun 2022 via PubMed. Keywords included nonalcoholic steatohepatitis with artificial intelligence, deep learning, and machine learning.

## 2. Results

### 2.1. NAFLD Diagnosis

Histologically, hepatic fat content is normal if the proportion of hepatocytes with fatty degeneration is below 5% [6,7,8,9]. If this number is over 5%, diagnosis of NAFLD will be considered when the possibility of other chronic liver diseases is ruled out [3,10]. Some invasive methods were widely applied in clinical practice. Less invasive methods that use serum biomarkers include fatty liver index (FLI) [11], hepatic steatosis index (HSI) [12], SteatoTest [13], and nonalcoholic fatty liver (NAFL) screening score [14]. The imaging methods were also widely used, including ultrasound [10], computed tomography (CT) [15], controlled attenuation parameter (CAP) [16], and magnetic resonance-based techniques [17]. Ultrasound is used as the first-line diagnostic tool to evaluate hepatosteatosis, and serum markers are alternative methods when non-invasive imaging technologies are unavailable in larger screening studies.

### 2.2. NASH Diagnosis

**Figure 1:**
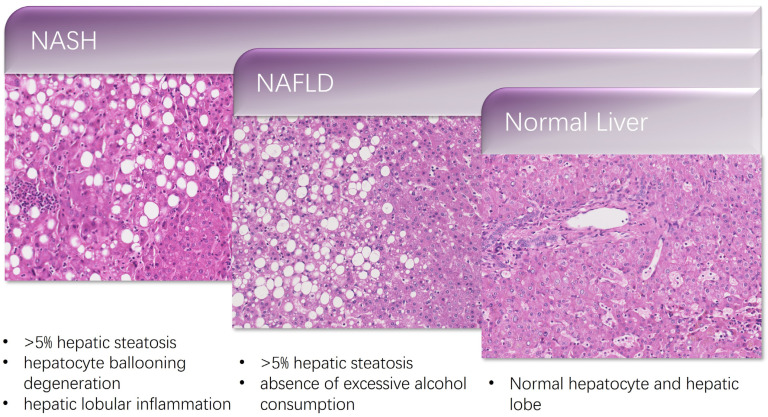
Histologic features of nonalcoholic steatohepatitis.

Nonalcoholic steatohepatitis (NASH) was first named in 1980 and indicates a situation of chronic liver inflammation [18]. The diagnosis of NAFLD requires evidence of hepatic steatosis of more than 5% by imaging or histology in the apparent absence of excessive alcohol consumption. By comparison, the diagnosis of NASH necessitates a biopsy, and the histological examination must show more than 5% hepatic steatosis, ballooning degeneration of hepatocytes, and inflammation of liver lobules (Figure 1). The system, NAFLD activity score (NAS), can be used to access the characteristics of NAFLD [19]. Numerous experimental settings in NAFLD studies have proven that characteristic-based NAFLD histological lesion scoring is effective.

NASH can be diagnosed without invasion by means of imaging, serum biomarkers, and biomarker panels. Common serum biomarkers include cytokeratin-18 (CK18) [20], inflammatory markers (CXCL10, TNF-α, IL-8) [21,22,23], adipocytokines and hormones (FGF21) [20], serum iron [24], etc. Biomarker panels include NASH Test [25], NASH ClinLipMet score [26], etc. Diagnostic imaging tools include vibration-controlled transient elastography (VCTE) [24], magnetic resonance elastography (MRE) [27], MRI-based technology assessing liver iron accumulation [24], multiparametric magnetic resonance imaging (MRI) technology [28], etc. Some of the above techniques demonstrate good sensitivity and specificity in small samples. In addition, some microRNAs, such as miR-34a and MiR-122 [28,29], also have the potential to be effective biomarkers.

### 2.3. NAFLD/NASH-Related Fibrosis

In the NAFLD/NASH population, there are four grades based on the degree of fibrosis. According to NASH-CRN, fibrosis has four categories: no fibrosis or mild fibrosis, significant fibrosis, advanced fibrosis, and cirrhosis [30]. The overall mortality rates of NAFLD patients in the last three categories are 1.6-, 3.04-, and 6.53-fold compared to that of nonfibrotic or mild fibrosis patients [31].

Commonly known biomarkers for predicting fibrosis are hyaluronic acid (HA), precursor C3-protein (PRO-C3), procollagen of type III collagen (PIIINP), and TIMP1 [32]. Some biomarker panels, such as FIB-4 [33], NAFLD fibrosis score (NFS) [34], and BARD score [35], were also applied to predict fibrosis. Several imaging tools were also widely used in the diagnosis of fibrosis. VCTE, an elastography modality performed by FibroScan using ultrasound-based technology, is the first FDA-approved elastography technique [36]. Shear wave elastography (SWE) [37], ARFI elastography [38], and MRE [39] were confirmed for diagnosis of liver fibrosis.

Biomarker panels are reproducible and cheap. They have a good NPV but have a low PPV. MRE has high accuracy for detecting fibrosis severity but is not widely used due to its high cost and low accessibility. Transient elastography, in addition to biomarker panels, is commonly used for evaluating the degree of fibrosis, but its efficiency should be further investigated in other independent studies. By combining serum biomarkers with imaging technologies, unnecessary liver biopsies can be largely reduced when detecting liver fibrosis.

### 2.4. Artificial Intelligence 

**Figure 2:**
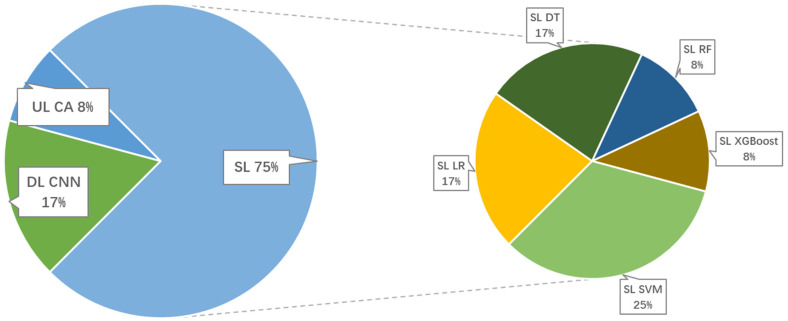
Percentages of applications of various ML techniques for NAFLD. SL: supervised learning; UL: unsupervised learning; DL: deep learning; LR: logistic regression; SVM: support vector machine; DT: decision tree; RF: random forest; CA: cluster analysis; CNN: convolutional neural network.

Artificial intelligence (AI) is a large field of transdisciplinary science. The scientific disciplines underlying AI include logic, statistics, cognitive psychology, decision theory, neuroscience, linguistics, cybernetics, and computer engineering. Machine learning (ML) is a subdiscipline of AI that enables computers to learn from data. ML is an overarching term for several methods to achieve AI and is the primary driver of the growth in AI commercial applications. ML has emerged as the chief AI tool to obtain cognitive insights, make predictions, and support decision making by a computer. ML represents a departure from earlier AI methods (expert systems) that operated by using an exhaustive set of logic rules, hand-coded in software, that attempted to anticipate all possible outcomes of a problem. With ML, computers can infer their own rules using advanced software methods (algorithms). It can be divided into three categories: supervised learning (the machine generates results by learning from both input and output data), unsupervised learning (the machine produces results without training of labeled data), and deep learning (the machine learns from a training dataset and predicts outcomes for new data). The current applications of AI in NAFLD and NASH are included in Table 1. Out of all 12 studies, 9 used supervised learning, 2 used unsupervised learning, and 1 used deep learning. Among them, the technique used for deep learning is the convolutional neural network; the technique used for unsupervised learning is cluster analysis; and the main techniques used for supervised learning are logistic regression, support vector machine, decision tree, random forest, and XGBoost. The percentage of use of each technique is shown in Figure 2. The support vector machine is the most applied technique in NAFLD study, accounting for 1/4 of all studies.

### 2.5. AI System Based on E-Health Record

Multi-disciplinary clinic models were recommended in the management of NAFLD [52,53]. Electronic health records (EHRs) record patient information, such as gender, BMI, ethnicity, laboratory test results, and comorbidities. Large datasets allow AI to detect risk factors of individual NASH patients.

In 2013, Douali N introduced a new clinical decision support system (CDSS) for diagnosing NASH and compared the system with machine learning algorithms. In this study, the accuracy of diagnosing NAFLD was 91.7% [40].

In 2018, Fialoke S used a machine learning method to predict NASH in NAFLD patients [41]. In this study, Optum Analytics, which included more than 80 million patients, was analyzed. Four machine learning models, logistic regression, decision tree, random forest, and XGBoost (all examples of supervised learning), were applied to create NASH classifiers, and 23 classifiers were confirmed. The best model was based on the XGBoost method and area under the receiver operating characteristic (AUROC) was 88%. This model was applied to a NAFLD cohort (N = 73,190); 45,797 patients were classified as NASH (62.6%) and 27,393 as healthy.

In 2019, NASHMap© (Novartis Pharma AG, Basel, Switzerland) was applied in real-world settings [42]. NASHMap© is used for predicting the occurrence of NASH based on 14 laboratory and clinical parameters. Various types of machine learning have advantages in interpreting each parameter to diagnose NASH. Among them, the XGBoost model works best [54]. This model adopted two large databases, the National Institute of Diabetes and Digestive and Kidney Diseases (NIDDK) registry and Optum® EMR, to legitimize the model. NASHMap© illustrated outstanding performance in the NIDDK dataset (AUROC of 0.82 and 0.80 in both 5- and 14-feature models, respectively), and the result was reproducible in the Optum® EMR dataset. NASHMap© successfully recognized that an extra number of 879,269 people had NASH who were not diagnosed in the Optum® EMR [42].

### 2.6. AI Based on Imaging

Ultrasound, CT, MRI, positron emission tomography, and histology are common medical imaging techniques. Supervised machine learning and deep learning algorithms can be tested on medical imaging. Random forests or support vector machines (SVM) (both examples of supervised ML) can use regions of interest (ROI) chosen by medical professionals or predefined information to identify image-based biomarkers on typical imaging. Deep neural networks can improve detection, classification, and segmentation accuracy. The convolutional neural network (CNN) has been the most widely used method deep learning. It can learn multiple convolutional filters and train classifiers simultaneously. It performs end-to-end learning to automatically extract desired characteristics. 

VCTE and ultrasound (US) elastography are common techniques with which to assess hepatic fibrosis. MRE can work not only more exactly but is also more capable of reproducing results than VCTE and US elastography. MRE can acquire more information to identify liver fibrosis, hepatic steatosis, and NASH if it is combined with MRI proton density fat fraction, which quantifies hepatic steatosis, and multiparametric MRI, which maps together fat fraction, liver stiffness, and fast T1 [43,44].

In 2019, Lili He a machine learning model that sorted out MRE-derived liver fibrosis according to the features of clinical and non-elastography MRI [45]. Support vector machine models practiced categorization by means of clinical features and radiomic features separately or with the use of both of them. The model internally assessed 225 patients and externally 84 patients in an independent cohort. In the internal cross-validation test, combined use of both features contributed the most remarkable result (AUROC = 0.84), whereas the other two results were clinical (AUROC = 0.77) or radiomic (AUROC = 0.70) features alone [45]. The combined feature allowed the SVM model to exactly assort patients with 81.8% accuracy, 72.2% sensitivity, and 87.0% specificity. In the external validation test, the SVM model generated 0.80 AUROC, 75.0% accuracy, 63.6% sensitivity, and 82.4% specificity [45]. This study demonstrated that this model, with the help of both clinical and T2-weighted radiomic features, can work quite well in the diagnosis of liver fibrosis.

In 2020, Schawk analyzed the diagnostic accuracy of liver fibrosis using parameters based on texture analysis (TA) by using MR elastrography with machine learning applied on T1w and T2w-phase images [46]. With 62 participants, TA and ML had accuracies of 85.7% on T1w and 61.9% on T2w in classifying high-grade and low-grade liver fibrosis. The AUC of TA in T1w was similar to that of the MRE, and the AUROC of the T2w phase was significantly lower than that of MRE. Schawkat’s study suggested that TA-derived measurements of T1w combined with ML have similar accuracy to that of the MRE in quantifying liver fibrosis. 

The above studies have proved that it is possible to achieve effective prediction of liver fibrosis by combining imaging and clinical data and applying machine learning methods, and suggests that non-invasive diagnosis of NASH is possible.

### 2.7. AI in Histology

In the NAS system, scores over four are defined representing clinical NASH. However, this system is semiquantitative due to the variation inter/intra-observer. At present, some AI attempts have been used to solve the above problems.

In 2018, Goh et al. reported the GENESIS system for the diagnosis and quantification of hepatic steatosis [47]. A new technology named second harmonic generation (SHG) microscopy uses multiphoton imaging techniques for histological tissue. In this study, microscopy analysis was performed on 86 preserved liver samples. The reliability of this study was supervised by three liver pathologists. 

In 2020, Liu et al. reported a qFIBS system for quantification of fibrosis, ballooning, steatosis, and inflammation for patients with NASH [48]. They used the second-harmonic-generation/two-photon excitation fluorescence technique to quantify specific histological patterns of NASH patients automatically. A qFIBS was established based on in silico analysis of four fundamental histological marked variables, which included inflammation (qInflammation), steatosis (qSteatosis), fibrosis (qFibrosis), and hepatocyte ballooning (qBallooning). Each variable was regarded as continuous but not categorical. Automated qFIBS analysis outputs showed a strong correlation with each element of the NASH Clinical Research Network scoring (*p* < 0.001; qFibrosis (*r* = 0.776), qBallooning (*r* = 0.533), qInflammation (*r* = 0.557), and qSteatosis (*r* = 0.802) ) and high AUROC values (qFibrosis (0.870–0.951; 95% confidence interval [1], [0.787–1.000], qBallooning [0.813–0.844], qInflammation [0.820–0.838), and qSteatosis [0.939–0.986]) [48]. The results showed a capability of distinguishing different stages of histological diseases.

In 2021, Taylor-Weiner et al. introduced another AI approach (PathAI) for quantifying liver histology and disease monitoring in NASH [49]. This system is based on a machine learning method to accurately measure NASH heterogeneity, severity, and treatment response. Histology samples were taken from three randomized controlled studies, and deep convolutional neural networks were used to validate major histological patterns in NASH, including inflammation, ballooning, steatosis, and fibrosis. This system generated reproducible and sensitive results, which suggests that machine learning can improve researchers’ acknowledgement of the disease development and heterogeneity of NASH, further categorize high risk patients, and improve the outcomes of NASH treatment in the long term. 

In addition, some studies of AI applications in histology have been reported. Teramoto T et al. used a topological data analysis methodology combined with linear ML techniques and applied this method using Matteoni classification to liver biopsies for stratifying NAFLD subtypes [50]. Forlano R et al. used ML to develop fully automated software for quantification of inflammation, steatosis, ballooning, and fibrosis in biopsy specimens from NAFLD patients and testified this method in a separate group of patients [51]. Data from 246 NAFLD patients with confirmed biopsy results were collected. The algorithm was trained by biopsy data of the first 100 subjects, and the training results were validated with the data of the remaining 146 samples. The computer-identified NAFLD histologic characteristics had an observer agreement of between 0.95 to 0.99. The results from the semiquantitative system scoring were from 0.58 to 0.88, lower than those of the computer identified features. In a paired liver biopsy specimen subgroup, quantitative analysis had an advantage in sensitivity in detecting distinctions compared to the NASH Clinical Research Network scoring system.

## 3. Conclusions

At present, AI is widely used in medical studies, especially in imaging diagnosis. With the increasing incidence of NAFLD, the diagnosis of NASH has become a major issue. The existing studies provide effective preliminary data support for the non-invasive diagnosis of NASH. However, it is still relatively difficult to obtain high-quality medical imaging data compared to big data from other industries. Data accumulated by a single medical institution are often insufficient to train an effective deep learning model, whereas those from different medical institutions are usually rarely interoperable and shared. In addition, training AI algorithms using medical images involves non-technical issues, such as protecting patient privacy. Therefore, there is considerable room for improvement in algorithms related to NASH diagnosis, including but not limited to relational analysis, quantitative (statistical) analysis, and hypothesis testing. Additionally, AI also requires the participation of medical institutions, medical experts, academic organizations, companies, and third-party operators to drive its development further. In the future, artificial intelligence promises to be an encouraging method to improve our ability to identify patients with NASH and those at risk for advanced fibrosis by objectively assessing liver images and improving deficiencies in the histological assessment of the liver. Artificial intelligence will be integrated into clinical care to aid in the care and follow-up of liver-related diseases. Based on larger cohorts, a NASH AI diagnosis system is likely to be developed and applied in clinical practice.

## Figures and Tables

**Table 1:** The current application of AI in NAFLD and NASH.

Data Type	Objective	Numbers of Patients	Features	Results	Country	Ref
e-health record	Predict NASH	162	Accuracy AUROC	Logistic regression gives an accuracy of 77.3% and Fuzzy Data Mining gives an accuracy of 75%. With CDSS, the accuracy was 91.9% and gives AUROC = 91.7%	France	[40]
Optum analytics	Diagnose NASH Predict NASH among NAFL patients	Total patients in database, *n* = 86 Mn NASH = 17,359 Healthy = 17,590 At risk NAFL = 73,190	Statistical summaries of temporal lab data (e.g., temporal mean) for ALT, AST, and PLT, basic demographics information and type 2 diabetes status	AUROC=83%-88% XG-Boost is the best Prevalence of NAFL cohort classified as NASH (48%) Prevalence of NAFL cohort classified as healthy (16%)	USA	[41]
NIDDK NAFLD adult database the Optum de-identified Electronic Health Record dataset	Diagnose NASH	Training set *n* = 704 Validation set *n* = 1016	NASHMap (HbA1c, AST, ALT, Total protein, AST/ALT, BMI, TG, Height, Weight, WBC, Hct, Alb, HTN, Gender)	XGBoost performed highest performance 14 and 5 feature model in NIDDK dataset (AUROC 82% and 80%) and Optum® EMR (AUROC of 76% and 74%)	USA Switzerland Germany	[42]
Imaging	Classify chronic liver disease (CLD)	126 patients (56 healthy controls, 70 with CLD)	ultrasound shear wave elastography (SWE) imaging, with a stiffness value clustering and machine learning algorithm	The highest accuracy in classification of healthy to CLD subject discrimination from the support vector machine model was 87.3% with sensitivity and specificity values of 93.5% and 81.2%, respectively. AUROC = 0.87 (confidence interval: 0.77–0.92)	Greece France USA	[43]
	diagnosis of fibrosis and NASH	104 consecutive adults	magnetic resonance elastography (MRE) ultrasound-based transient elastography (TE) liver biopsy analysis	MRE detected any fibrosis with AUROC = 0.82 MRI-based proton density fat fraction (MRI-PDFF) identified steatosis of grades 2 or 3 with AUROC =0.90	USA	[44]
	diagnosis of liver disease	internally evaluated in 225 patients (mean age, 14.1 years) and externally evaluated in 84 patients (mean age, 13.7 years)	Clinical and T2-Weighted MRI Radiomic Data	The combination of clinical and radiomic features produced the best performance (AUC = 0.84), compared with clinical (AUC = 0.77) or radiomic (AUC = 0.70) features alone. Support vector machine (SVM) model in external validation with an accuracy of 75.0%, sensitivity of 63.6%, specificity of 82.4%, and AUC of 0.80.	USA	[45]
	liver fibrosis quantification	62 consecutive participants	Texture analysis (TA) –derived parameters combined with machine learning (ML) of non-contrast-enhanced T1w and T2w fat-saturated (fs) images vs MR elastography (MRE)	The AUC for TA on T1w was similar to MRE (0.82 [95% CI 0.59–0.95] vs. 0.92 [95% CI 0.71–0.99], *p* = 0.41)	Switzerland	[46]
Histology	Quantifying hepatic steatosis (HS)	86 archived liver biopsy samples	Second Harmonic Generation microscopy analysis using GENESIS (HistoIndex, Singapore)	Good correlation was observed between the histopathologists and automated SHG microscopy assessment of HS with Pearson correlation of 0.93: *p* < 0.001	Singapore	[47]
	Quantitative Evaluation of Fibrosis, Inflammation, Ballooning, and Steatosis in Patients With NASH	219 nonalcoholic fatty liver disease (NAFLD) /NASH liver biopsy samples	qFIBS, a computational algorithm that quantifies key histological features of NASH	Automated qFIBS analysis outputs showed strong correlation with each respective component of the NASH CRN scoring (*p* < 0.001; qFibrosis [r = 0.776], qInflammation [*r* = 0.557], qBallooning [*r* = 0.533], and qSteatosis [*r* = 0.802]) and high area under the receiver operating characteristic curve values (qFibrosis [0.870–0.951; 95% confidence interval {CI}, 0.787–1.000; *p* < 0.001], qInflammation [0.820–0.838; 95% CI, 0.726–0.933; *p* < 0.001), qBallooning [0.813–0.844; 95% CI, 0.708–0.957; *p* < 0.001], and qSteatosis [0.939–0.986; 95% CI,0.867–1.000; *p* < 0.001])	China Singapore United Kingdom	[48]
	Quantitative measurement of liver histology and disease monitoring	liver biopsy samples from three randomized controlled trials of therapies for patients with advanced fibrosis attributable to NASH (STELLAR-3 [NCT03053050], STELLAR-4[NCT03053063], and ATLAS [NCT03449446]).	PathAI (machine learning-based approach)	ML method has shown reproducibility and sensitivity and was prognostic for disease progression, demonstrating the power of ML to advance our understanding of disease heterogeneity in NASH, risk stratify affected patients, and facilitate the development of therapies	USA	[49]
	Differentiate between non- NASH and NASH	79 NAFLD patients	topo- logical data analysis methodology combined with linear machine learning techniques	Over 90% accuracy for the classification between NASH and non-NASH NAFLD groups. AUROC 0.946 for the classification of NASH and NAFL2 (type 2 of Matteoni classification),	Japan	[50]
	quantification of steatosis, inflammation, ballooning, and fibrosis in biopsy specimens from patients with NAFLD	246 consecutive patients with biopsy-proven NAFLD	machine learning	software identified histologic features of NAFLD interobserver and intraobserver agreement (0.95–0.99); higher than semiquantitative scoring systems, (0.58–0.88)	United Kingdom Greece	[51]

## Data Availability

Not applicable.
